# Modulation of Lipid Transport and Adipose Tissue Deposition by Small Lipophilic Compounds

**DOI:** 10.3389/fcell.2020.555359

**Published:** 2020-10-14

**Authors:** José M. Castellano, Juan M. Espinosa, Javier S. Perona

**Affiliations:** Group of Bioactive Compounds, Nutrition and Health, Department of Food and Health, Instituto de la Grasa-Consejo Superior de Investigaciones Científicas, Seville, Spain

**Keywords:** tocopherol, carotenoid, sterol, triterpene, metabolism, metabolic disease

## Abstract

Small lipophilic molecules present in foods of plant origin have relevant biological activities at rather low concentrations. Evidence suggests that phytosterols, carotenoids, terpenoids, and tocopherols can interact with different metabolic pathways, exerting beneficial effects against a number of metabolic diseases. These small molecules can modulate triacylglycerol absorption in the intestine and the biosynthesis of chylomicrons, the lipid carriers in the blood. Once in the bloodstream, they can impact lipoprotein clearance from blood, thereby affecting fatty acid release, incorporation into adipocytes and triglyceride reassembling and deposit. Consequently, some of these molecules can regulate pathophysiological processes associated to obesity and its related conditions, such as insulin resistance, metabolic syndrome and type-2 diabetes. The protective capacity of some lipophilic small molecules on oxidative and chemotoxic stress, can modify the expression of key genes in the adaptive cellular response, such as transcription factors, contributing to prevent the inflammatory status of adipose tissue. These small lipophilic compounds can be incorporated into diet as natural parts of food but they can also be employed to supplement other dietary and pharmacologic products as nutraceuticals, exerting protective effects against the development of metabolic diseases in which inflammation is involved. The aim of this review is to summarize the current knowledge of the influence of dietary lipophilic small biomolecules (phytosterols, carotenoids, tocopherols, and triterpenes) on lipid transport, as well as on the effects they may have on pathophysiological metabolic states, related to obesity, insulin resistance and inflammation, providing an evidence-based summary of their main beneficial effects on human health.

## Introduction

Among lipophilic compounds exerting biological effects on human health, phytosterols, carotenoids, tocopherols, and triterpenes are consumed as part of food. To exert their benefit, they need to be released from the food matrix and be available for intestinal absorption (Saura-Calixto et al., 2007); this involves micelle formation and emulsification by bile, interaction with enzymes in the intestinal lumen, and further hydrolysis by pancreatic lipases, if they were esterified with fatty acids (Iqbal and Hussain, 2009).

Latest studies using the human epithelial colorectal adenocarcinoma (CaCo-2) cell line have proved that the uptake of these substances is mediated by transporters, being therefore a saturable process (During et al., 2002; Anwar et al., 2006; Amiot et al., 2011). Through different mechanisms, that will be detailed onward, once these lipophilic compounds arrive in the enterocyte, they need to be incorporated into chylomicrons, so they can be transported in the blood and to the lymph, both aqueous mediums, ending up in the liver and extrahepatic tissues.

Carotenoids and tocopherols are transported in the core of chylomicrons (Iqbal and Hussain, 2009) but less is known about sterols or triterpenoids. For instance, we reported that oleanolic acid (OA) is transported in plasma bound to albumin, but it cannot be discarded that this triterpene can also be transported in lipoprotein carriers (Rada et al., 2015).

Phytosterols, carotenoids, tocopherols, and triterpenes can exert important biological effects even before arriving at the target tissues. There is evidence that they could modulate triacylglycerol (TAG) hydrolysis by lipoprotein lipase (LPL) in chylomicrons (Cabello-Moruno et al., 2014), which affect their clearance from plasma and liver uptake (Perona et al., 2006), and improve the balance between vasoprotective and prothrombotic factors (Perona et al., 2004). Therefore, they have therapeutic potential to be used against chronic metabolic diseases related to TAG transport and deposition in adipose tissue.

Below, we summarize the current knowledge of the influence of these dietary lipophilic small biomolecules on lipid transport, as well as on the effects they may have on pathophysiological metabolic states related to obesity, insulin resistance and inflammation.

## Phytosterols

### Definition, Types and Structure

**FIGURE 1:**
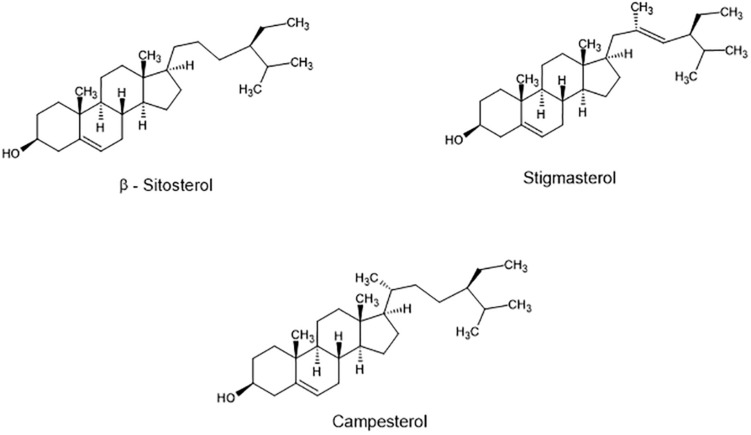
Structure of main phytosterols found in food.

Phytosterols are bioactive components present in plants that are synthesized via the isoprenoid pathway, while phytostanols are their saturated derivatives. Their main function is to stabilize plant cell membranes and serve as precursors in the synthesis of steroidal saponins, alkaloids, and other steroids (Lai and Akoh, 2005). These plant sterols share structural similarity with cholesterol, differing in a methyl or ethyl group in C24. The most abundant sterols in plants and plant-containing foods are sitosterol (C_29_H_50_O), campesterol (C_29_H_48_O), and stigmasterol (C_29_H_48_O) (Figure 1), accounting for about 90% of total sterols to the diet (Klingberg et al., 2008).

### Dietary Sources and Bioavailability

The main sources of plant sterols are vegetable oils, nuts and unrefined grains, whereas plant stanols are mainly present in cereals, especially wheat and rye (Valsta et al., 2004). Other important dietary sources are phytosterol-enriched food products, usually in their esterified form, including margarines, yogurts and beverages.

Absorption of phytosterols follows the same pathways as cholesterol in the proximal part of the small intestine. Free sterols are solubilized into the micelle that is formed in the emulsified fat phase. In a group of ten healthy subjects, it was found that phytosterols are absorbed in the brush border membrane of the enterocyte via transporter proteins, such as Niemann-Pick C1-Like 1 (NPC1L1) with a very low efficiency (<2% for sterols and <0.2% for stanols) compared to cholesterol (Ostlund et al., 2002). Consequently, their serum concentrations are low, varying from 7 to 24 mmol/L for sterols, and from 0.05 to 0.3 mmol/L for stanols.

### Effects on Lipid Absorption and Transport

Phytosterols have the ability to modulate serum cholesterol transport and metabolism. The FDA and EFSA have approved health claims for functional foods that provide 1.3 g (FDA, 2010) or 3.0 g (EFSA, 2015) of plant sterols/day respectively for reducing serum total cholesterol (TC) and LDL (Brufau et al., 2008; Musa-Veloso et al., 2011). Nevertheless, the clinical relevance of these cholesterol lowering effects is still a matter of controversy (Talati et al., 2010). The impairment in cholesterol absorption by displacement from micelles in the intestinal lumen by phytosterols has been suggested as the underlying mechanism (Amiot et al., 2011), however, there is also evidence that they modify the expression of genes involved in cholesterol re-esterification in the enterocyte and its removal via trans-intestinal cholesterol efflux (Gylling and Simonen, 2015).

Phytosterols may likewise contribute to reduce serum TAG (Demonty et al., 2013) by decreasing their intestinal absorption (Rideout et al., 2010), restricting chylomicron assembly in the enterocyte (Liang et al., 2011) and reducing the hepatic release of very-low-density lipoproteins (VLDL) (Gylling and Miettinen, 1994). Reductions in serum TAG levels of 6–20% by the intake of 1.5–2 g/day of phytosterol/phytostanols have been reported (Sialvera et al., 2012; Demonty et al., 2013; De Smet et al., 2015). However, other studies did not corroborate such changes (Amiot et al., 2011; De Smet et al., 2015).

### Implications in Metabolic Diseases

Metabolic syndrome (MetSyn) is a cluster of several pathophysiological states, including central obesity, hyperglycemia, hypertriacylglycerolemia, hypertension and low HDL, which may increase the risk of type 2 diabetes (T2DM), cardiovascular disease (CVD), neurodegenerative disorders and certain types of cancer (Mottillo et al., 2010). There are data indicating that consumption of phytosterols may have beneficial effects on MetSyn subjects (Rondanelli et al., 2013; Coker et al., 2015), although controversy remains (Ooi et al., 2007). A 16-weeks study in gestational diabetic women taking phytosterol-rich margarine reported an increase in serum HDL and improvements in markers of glucose homeostasis, including fasting glycemia, fasting insulin, HOMA-IR (homeostatic model assessment for insulin resistance) and β-cell function (Li and Xing, 2016). In contrast, a randomized controlled trial (RCT) with 151 T2DM patients taking a low-fat spread enriched in phytosterols (2 g/day) for 6 weeks reported reductions in serum TAG and LDL, but no effects on postprandial glycemia (Trautwein et al., 2018).

Both *in vitro* (Kurano et al., 2011; Valerio and Awad, 2011) and *in vivo* (mice and piglets experimental models) (Hu et al., 2017; Plat et al., 2014) experiments indicate that phytosterols exhibit anti-inflammatory properties. However, systematic reviews and meta-analyses of RCT do not support that the regular intake of phytosterol-enriched foods reduced low-grade systemic inflammation associated to obesity (Rocha et al., 2016).

## Carotenoids

### Definition, Types and Structure

**FIGURE 2:**
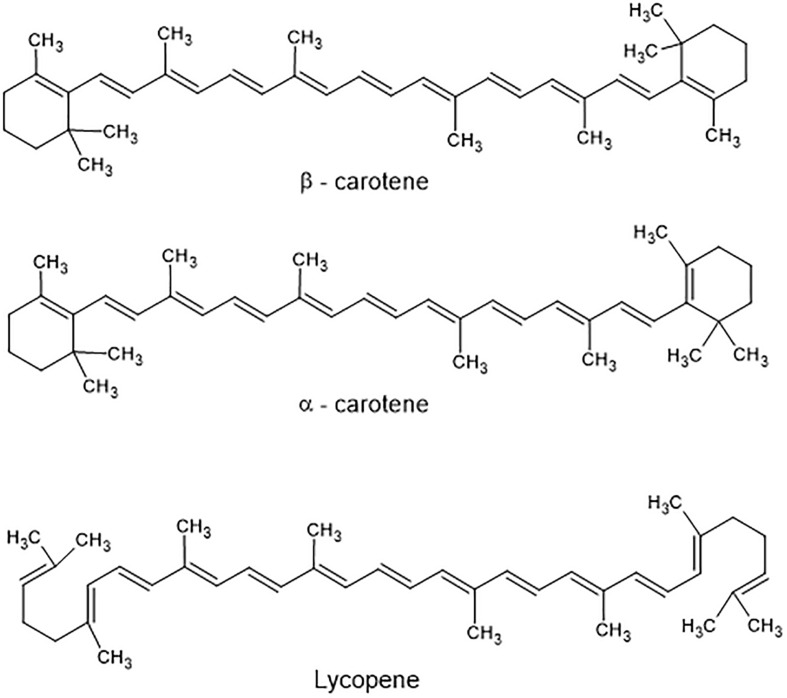
Structure of main carotenes found in food.

**FIGURE 3:**
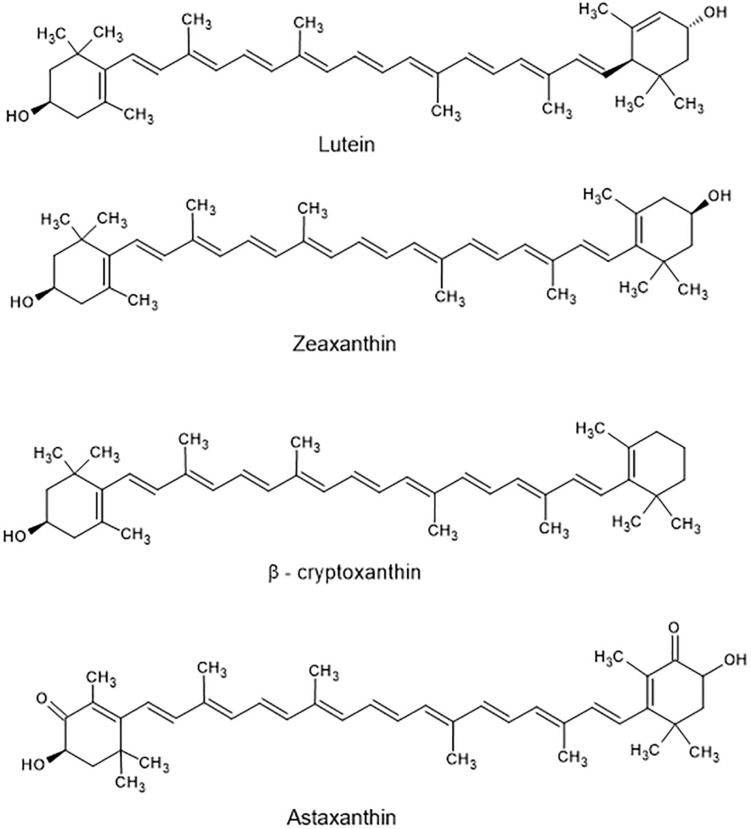
Structure of main xanthophylls found in food.

Carotenoids include both carotenes and xanthophylls. They are poly-unsaturated isoprenoids, often consisting of eight isoprene units. Thus, many carotenoids belong to the tetraterpenes. At the ends of the carbon chain various functional groups can be located, resulting in the enormous variety of more than 750 carotenoids known today (Westphal and Böhm, 2015). Carotenes harbor hydrocarbon-type structures (Figure 2), whereas xanthophylls contain oxygen in their molecules (Figure 3). Although more than one thousand carotenoids have been identified (Yabuzaki, 2017), only about 40 are present in human blood and tissues. β-Carotene (C_40_H_56_), α-carotene (C_40_H_56_), and lycopene (C_40_H_56_) are the main carotenes, while lutein (C_40_H_56_O_2_), zeaxanthin (C_40_H_56_O_2_), β-cryptoxanthin (C_40_H_56_O), and astaxanthin (C_40_H_52_O_4_) are the main xanthophylls (Figure 2).

### Dietary Sources and Bioavailability

β-carotene shows the greatest capability to be incorporated into mixed micelles, while that of lycopene is very poor (Sy et al., 2012). In fact, and unlike other carotenoids, lycopene levels in plasma and tissues do not correlate well with its dietary intake (Gann, 2005). According to The Carotenoid Content of US Foods (Holden et al., 1999) and data from O’Neill et al. (2001) the most abundant food sources of carotenoids in the United States and Europe are carrot for α and β-carotene, tomato and watermelon for lycopene, kale and parsley for lutein, red pepper for zeaxanthin and papaya for β-cryptoxanthin.

Experiments in Caco-2 cells show that carotenoid uptake is curvilinear, time-dependent, saturable and dose-dependent (During et al., 2002), being facilitated by epithelial transporters with broad substrate specificity (Reboul and Borel, 2011), such as the scavenger receptor B1 (SR-B1) (During et al., 2005). After intestinal absorption, carotenoids are incorporated into chylomicrons, which account for 80% of total plasma carotenoids in the postprandial period, and transported to the liver, their main storage organ (During et al., 2002).

### Effects on Lipid Absorption and Transport

In rats fed a high-fat diet, lycopene-enriched tomato juice reduced plasma and hepatic TAG (Kim et al., 2012). Moreover, this juice dose-dependently decreased serum TAG, TC and LDL in hypercholesterolemic hamsters (Lee et al., 2015). In humans, the meta-analysis of twelve intervention studies (Ried and Fakler, 2011) pointed out that supplementation with lycopene-rich edible sources (≥25 mg lycopene/day) reduced LDL by about 10%. However, a more recent meta-analysis (Cheng et al., 2017) reported that tomato supplements successfully reduced LDL, while supplementation with lycopene alone yielded no significant effects. Plasma levels of α-carotene, β-cryptoxanthin, lutein, zeaxanthin, and lycopene, but not β-carotene, have showed positive correlations with plasma TC concentrations (Amara et al., 2015). Likewise, β-cryptoxanthin, lutein, zeaxanthin, and lycopene also positively correlated with LDL. β-Carotene, β-cryptoxanthin, lutein, and zeaxanthin further did it with HDL. A trial with 670 non-diabetic Mexican-American children determined negative correlations of α-/β-carotene with TAG and positive associations with HDL (Farook et al., 2017). By contrast, a research associated to the CARET Study (Omenn et al., 1996) showed that the combined administration of β-carotene and retinyl palmitate to smoker or asbestos-exposed individuals did not produce significant differences in plasma TAG, TC, and LDL when compared with those receiving placebo (Redlich et al., 1999).

The plasma TAG lowering effect of lycopene has been explained by mRNA overexpression of LPL and increased TAG hydrolysis, but also by enhanced hepatic fatty acid β-oxidation (Martín-Pozuelo et al., 2015).

The most important effect of carotenoids on lipids and proteins is their ability to protect them from oxidation. The daily administration of 280 mL of tomato juice (containing 32.5 mg of lycopene) to young females for 2 months decreased plasma levels of malondialdehyde (MDA), a maker of lipid peroxidation, in parallel with a reduction in body fat (Li et al., 2015). However, this was an uncontrolled supplementation trial, so results should be taken with caution. Nevertheless, carotenes seem to be worse superoxide radical quenchers than xanthophylls, especially than those containing carbonyls, like canthaxanthin and astaxanthin (Galano et al., 2010).

### Implications in Metabolic Diseases

Different studies have consistently described an inverse association of the carotenoid dietary intake with BMI, insulin resistance, MetSyn and CVD (Czernichow et al., 2009; Sluijs et al., 2009; Chai et al., 2010; Suzuki et al., 2011; Higuchi et al., 2015). In adipose tissue, carotenoids influence signaling pathways and gene expression which modulate the pro-inflammatory cytokines secretion and the proliferation/differentiation of adipocytes (Sy et al., 2012; Östh et al., 2014). In mice, a β-carotene-enriched diet decreased body weight, fat mass and adipocyte size, through the PPARα-mediated overexpression of β-carotene-15,15′-oxygenase (Amengual et al., 2011). Also in mice, lycopene restricted adipocyte hypertrophy caused by high-fat diets (Fenni et al., 2017).

In MetSyn patients, carotenoid intake correlated with reductions in waist circumference, visceral fat and subcutaneous fat mass (Sluijs et al., 2009). Carotenoid treatments have been also associated with the improvement of insulin signaling. An inverse association between carotenoids and HOMA-IR was established (Suzuki et al., 2011; Farook et al., 2017; Xiao et al., 2019), although this correlation was not conserved in adjusted models for BMI and waist circumference (Amara et al., 2015). The insulin-sensitizing effect of carotenoids has been attributed, at least partially, to their ability to enhance adiponectin secretion by adipose tissue (Amara et al., 2015; Li et al., 2015; Grasa-López et al., 2016) and to increase the insulin receptor substrate-2 (IRS-2) expression in the liver (Awazawa et al., 2011).

## Tocopherols and Tocotrienols

### Definition, Types and Structure

“Vitamin E” refers to hydroxychromane derivatives with antioxidant activities. The most common forms of vitamin E are tocopherols (TP) and tocotrienols (T3), but there are also tocomonoenols (T1) and marine derived tocopherols (MDT). Usually, vitamin E is generically named “tocopherols.” Their basic structure is a chromium ring hydroxylated at position 6, the methylation of which classifies them into α, β, γ, or δ forms. Four families are differentiated by differently saturated side chains (Table 1). These vitamers are naturally in *all R* configuration (Traber and Atkinson, 2007).

**TABLE 1:** Common name, structure, and formula of the four vitamin E families.

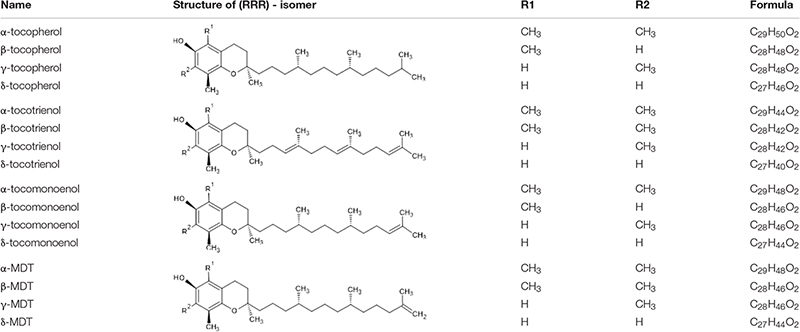

### Dietary Sources and Bioavailability

Vitamin E is found in food such as vegetable oils (sunflower, palm, olive, cocoa, safflower, grape seed), soy beans, olives, blueberries, nuts, grains (wheat, rice, barley) and culinary herbs (cloves, cumin) (Aggarwal et al., 2010). When consumed, tocopherols dissolve into the meal lipid phase and are emulsified at the stomach and duodenum as mixed micelles. Micelles are dissociated and tocopherols absorbed by enterocytes in the intestinal brush border, by both passive diffusion and mediated by receptors, such as the scavenger receptor class B type I (SR-BI) (Reboul et al., 2006) NPC1 like intracellular cholesterol transporter 1 (NPC1L1) (Reboul et al., 2012), and CD36 (Goncalves et al., 2014). Only free forms of vitamin E seem to be uptaken, suggesting that esterified forms are hydrolyzed beforehand (Lombardo and Guy, 1980). Once absorbed, tocopherols integrate in chylomicrons, which are first released to the lymph, and subsequently to the bloodstream. A fraction of tocopherols in chylomicrons is captured by extrahepatic tissues (mainly adipose tissue), whereas the rest goes to the liver in remnant chylomicrons. In addition, tocopherols transfers from chylomicrons to HDL (Traber et al., 2019) and from LDL to HDL (Mardones and Rigotti, 2004) so occur. They are important for vitamin E delivery to reproductive tissues (adrenals, ovaries, and testes), lung and brain. In the liver, α-tocopherol is specifically bound to α-TF transfer protein (α-TTP), which protects it from catabolism, allowing its incorporation into nascent VLDL (Mustacich et al., 2007). The excess of α-TF and other vitamers are secreted in bile or metabolized to carboxyethyl-hydroxychroman (CEHC) and later excreted in urine (Schultz et al., 1995).

Dietary lipids are effectors of vitamin E absorption. MUFA and PUFA promoted tocopherols absorption compared to saturated ones in cockerels (Prévéraud et al., 2014) and Caco-2 cells (Failla et al., 2014). Conversely, phosphatidylcholine, vitamin C, carotenoids, and polyphenols significantly impair it in rats (Koo and Noh, 2001; Nishimukai and Hara, 2004; Reboul et al., 2007; Goncalves et al., 2015). There is controversial data about the fat quantity required for optimal vitamin E absorption (Roodenburg et al., 2000; Bruno et al., 2006). A recent trial with healthy women has determined that α-TP absorption is not quantitatively limited by the dietary fat abundance or fasting (Traber et al., 2019). The authors pointed that α-TP is retained in the enterocyte until sufficient fat is consumed to promote chylomicron secretion.

### Effects on Lipid Absorption and Transport

α-TP diminishes the capture of ox-LDL by monocytes/macrophages through CD36 downregulation (Ricciarelli et al., 2000; Munteanu et al., 2006).

Analogously, T3 decrease serum NEFA, TAG, TC, LDL, Apo B, glucose and HbA1c levels and hepatic cholesterol (Qureshi et al., 2000; Chou et al., 2009), while increase HDL (Budin et al., 2009; Matough et al., 2014). T3 decline likewise HMG-CoA reductase and hyperlipidemia in murine models of hypercholesterolemia and atherosclerosis (Iqbal et al., 2003; Minhajuddin et al., 2005).

### Implications in Metabolic Diseases

Tocopherols are potent antioxidants within lipid domains, both *in vitro* (fats and food oils) and *in vivo* (biological membranes, lipoproteins and tissues) (Kuhad and Chopra, 2009; Siddiqui et al., 2010; Wong et al., 2017). α-TP efficiently decreased hydroxyl and superoxide radicals and scavenged peroxides in different animal models (Cachia et al., 1998; Alcalá et al., 2015) and increased NO production (Meydani et al., 2014). γ-TP attenuated superoxide, lipid peroxides and ox-LDL in arteries of Sprague Dawley rats (Saldeen et al., 1999). It significantly increased NOS activity and plasma nitrites and also enhanced endogenous SOD and glutathione peroxidase activities in spontaneously hypertensive rats (SHR) (Newaz et al., 2003; Budin et al., 2009; Matough et al., 2014). T3 reduced lipid peroxidation and oxidative stress in murine models (Kuhad and Chopra, 2009; Burdeos et al., 2012) and HepG2 cells (Asai et al., 1999). These antioxidant response is consistent with the vitamin E role as Nrf2 activator (Bozaykut et al., 2014). In experimental animals, α-TP improved hypertriglyceridemia, insulin resistance and hepatic steatosis (Alcalá et al., 2015).

Vitamin E also performs against inflammation; α-TP inhibited PKC, 5-LOX and PLA2, and activated PP2A and DAG kinase (Mathur et al., 2015; Hayashi et al., 2017). It repressed the activation of nuclear factor κB (NFκB), as well as the biosynthesis of pro-inflammatory cytokines and adhesion molecules (Cook-Mills, 2013; Rashidi et al., 2017). Likewise, γ-TP blocked COX activity and diminished prostaglandin E2 (PGE2) synthesis (Jiang et al., 2000; Yoshikawa et al., 2005). γ-T3 inhibited the production of TNF-α, transforming growth factor-beta (TGF-β), and IL-1β in STZ-diabetic rats. In human adipocytes, γ-T3 suppressed MAP kinase and NFκB pathways (Kuhad et al., 2009). In C57BL/6J mice, γ-T3 improved insulin signaling and glucose tolerance. It also decreased MCP-1 in adipose tissue, indicating a lesser macrophage infiltration (Zhao et al., 2015). Despite these beneficial effects, some authors have suggested that high doses of vitamin E could become harmful. For instance, a dose of 600 mg α-TP/kg augmented blood pressure and lipid peroxides in serum and brain tissue of SHR (Miyamoto et al., 2009).

In humans, evidence of vitamin E effects on metabolic disorders is still not solid. Some trials showed that vitamin E improves dyslipidemia in patients of MetSyn (Devaraj et al., 2008; Heng et al., 2015), hypercholesterolemia (Qureshi et al., 2002; Zaiden et al., 2010), or diabetes (Baliarsingh et al., 2005). Furthermore, tocopherols improved glycemic control (Irandoost et al., 2013), hypertension (Jain and Jain, 2012) and increased the endogenous antioxidant capacity (Vafa et al., 2015). In animal models, α-TP improves hypertriglyceridemia, insulin resistance and hepatic steatosis (Alcalá et al., 2015). It also reduces LXRα expression and increases ABCA1, preventing cholesterol-mediated damage to cardiomyocytes (Sozen et al., 2018).

Cohort studies and RCT have displayed an inverse association between vitamin E and the risk of ischemic cardiomyopathy (Venturi et al., 2019), stroke (Boaz et al., 2000), coronary artery disease (Muntwyler et al., 2002), myocardial infarction (Stephens et al., 1996; Boaz et al., 2000) and death due to heart failure (Muntwyler et al., 2002; Eshak et al., 2018). However, other well-designed trials, such as SU.VI.MAX (Kubota et al., 2011) and PREDIMED (Henríquez-Sánchez et al., 2016), did not determine any relationship between vitamin E supplementation and CVD incidence and mortality. Neither on circulating lipids (O’byrne et al., 2000; Mustad et al., 2002; Rasool et al., 2006).

## Pentacyclic Triterpenes

### Definition, Types and Structure

**FIGURE 4:**
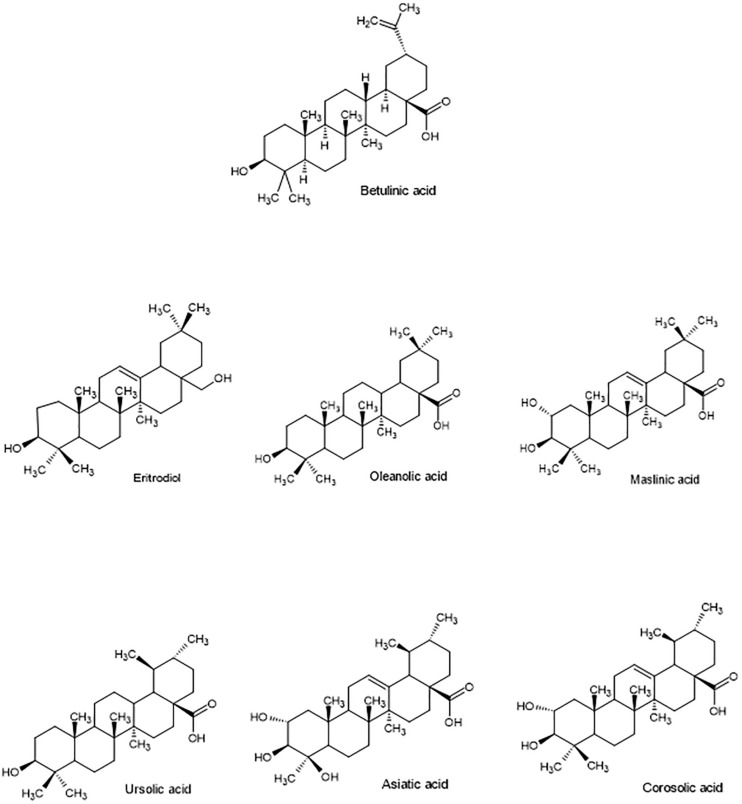
Structure of main bioactive pentacyclic triterpenes of lupine, oleanane and ursane subtypes.

Pentacyclic triterpenes (PT) are synthetized, as phytosterols, through the mevalonate pathway and oxidosqualene cyclization. Most frequent PT belong to three subtypes: lupane (betulinic acid), oleanane (uvaol, erythrodiol, oleanolic and maslinic acids) and ursane (ursolic, asiatic, corosolic, and boswellic acids) (Figure 4). PT can occur as free, acylated (with hydroxycinnamic acids or fatty acids, for instance) or glycosylated (triterpenoid saponins) forms (Furtado et al., 2017).

### Dietary Sources and Bioavailability

Pentacyclic triterpenes are natural components of a great variety of fruits, vegetables, and medicinal plants and are therefore part of the human diet. They are found in apple, pear, mango, green pepper, strawberries, mulberry, guava or olives; but also in aromatic herbs (e.g., basil, oregano, rosemary, and lavender) (Jäger et al., 2009). *In vivo* studies have shown that PT bioavailability differs when they are administered as pure compounds or in a complex matrix, such as a food item. The presence of fat appears also of major importance, since the solubilization and micellarization of lipophilic compounds are necessary steps prior to absorption (Furtado et al., 2017). Although absorption and metabolism processes are not well established, both passive and *P*-glycoprotein-mediated active transport have been postulated for intestinal absorption of PT (Wang et al., 2017; Jinhua, 2019). They result widely distributed among tissues after passing through the liver (Rada et al., 2011; Zhu et al., 2013).

### Effects on Lipid Absorption and Transport

In animal models of dyslipidemia, PT reduce plasma TAG, TC, LDL, VLDL and NEFA, whereas significantly increase HDL and NO (Wang et al., 2013; Pan et al., 2018), enhance plasma leptin and reduce ghrelin (Wang et al., 2013). They downregulate lipogenic genes (ACC, SCD2, GPAT3, ACAT) (Wang et al., 2013) and diminish hepatic fatty acid oxidation by downregulating PPAR-γ coactivator-1β (PGC-1β) (Kuroda et al., 2012). In addition, PT regulate the expression of genes involved in regulation of lipid metabolism, such as PPAR-α (Quang et al., 2011), PPAR-γ (Luo et al., 2018), *AdipoR1*, and *AdipoR2* (Sung et al., 2010).

### Implications in Metabolic Diseases

Pentacyclic triterpenes present several other biological activities, including anti-inflammatory, antioxidant, anti-viral, anti-diabetic, anti-tumor, hepatoprotective, and cardioprotective, and could be used as anti-ulcer drugs, as well as for the prevention and treatment of metabolic diseases (Reen-Yen et al., 2009; Yamai et al., 2009; de Melo et al., 2010; Jiang et al., 2015; Fernández-Aparicio et al., 2019). As a result, some triterpenes are currently being evaluated in clinical trials (Skarke et al., 2012; Santos-Lozano et al., 2019).

On peripheral tissues, PT improve insulin signaling, upregulating the expression of IR and IRS (Whalley et al., 2011), promoting IR auto-phosphorilation (Sato et al., 2007) and selectively inhibiting tyrosine phosphatases PTP1B and TCPTP (Jung et al., 2007; Teodoro et al., 2008; Genet et al., 2010; Bu et al., 2011). Moreover, PT arise the activity of essential kinases for insulin-stimulated events, such as the PI3K/Akt axis (Galic et al., 2005; Ramírez-Espinosa et al., 2011), ERK 1/2, LKB1, and AMPK (Feng et al., 2011). In the liver, PT inhibit GSK3β (Sangeetha et al., 2010; Zeng et al., 2012; Ramírez-Espinosa et al., 2011) and potentiate the glycogen pool through the stimulation of glucokinase activity and the repression of glucose-6-phosphatase and glycogen phosphorylase (Ha et al., 2009; Azevedo et al., 2010; Saha et al., 2010). Another hypoglycemic effect of PT is their ability to strongly inhibit intestinal and pancreatic α-glucosidases (Ali et al., 2002; Castellano et al., 2016). Likewise, PT markedly reduce microvesicular steatosis and lipid droplets in the liver (Woo et al., 2006).

PT inhibit the polyol pathway and attenuate the synthesis of advanced glycation end-products (AGEs). They inhibit aldose reductase and sorbitol dehydrogenase (Cheng et al., 2010), and enhance glyoxalase-I. In rodent, they reduce the formation of methylglyoxal, pentosidine, *Nε*-(carboxymethyl)lysine (Ahn et al., 2017), plasma HbA1c and urinary glycated albumin (Bachhav et al., 2015).

Although modest radical scavengers (Li et al., 2014; Wang et al., 2015; Castellano et al., 2016; Lee et al., 2016), PT strongly potentiate the adaptive cell response against oxidative and chemotoxic stresses. They stimulate the expression of antioxidant and NADPH-producing enzymes (Djeziri et al., 2018; Gamede et al., 2018; Su et al., 2018), and reduce LDH and MDA productions (Djeziri et al., 2018). In these effects, the activation of the nuclear factor Nrf2 seems to play a key role (Yin and Chan, 2007; Allouche et al., 2011; Castellano et al., 2013).

Furthermore, PT block NFκB activation (Belleza et al., 2010; Castellano et al., 2013), and irreversibly inhibit phospholipase A2 (Dharmappa et al., 2009), attenuating the production of pro-inflammatory cytokines (Du and Ko, 2006; Yang et al., 2007; Tsai and Yin, 2008). PT enhance the levels of angiotensin 1-7, NO and eNOS (Soobrattee et al., 2005). In experimental animals, PT decrease hepatic and adipose tissue productions of ROS, IL-1b, IL-6, IL-18, and TNFα (Huang et al., 2005; Chen et al., 2006, 2017; Wang et al., 2010; Saaby et al., 2011), together with the inhibition of NLRP3 inflammasome and caspase-1 pathways (Wang et al., 2010).

At β-cell level, PT increase the glucose-stimulated insulin biosynthesis and secretion through a multifactorial mechanism. They stimulate pro-insulin gene expression (Gilon and Henquin, 2001), activate M3-subtype muscarinic receptors (Ali et al., 2002), and perform as selective agonists of TGR5 receptors (Genet et al., 2010). PT act likewise as anti-apoptotic agents and selective enhancers of the Shp-2 phosphatase activity (Ali et al., 2006).

## Concluding Remarks

**FIGURE 5:**
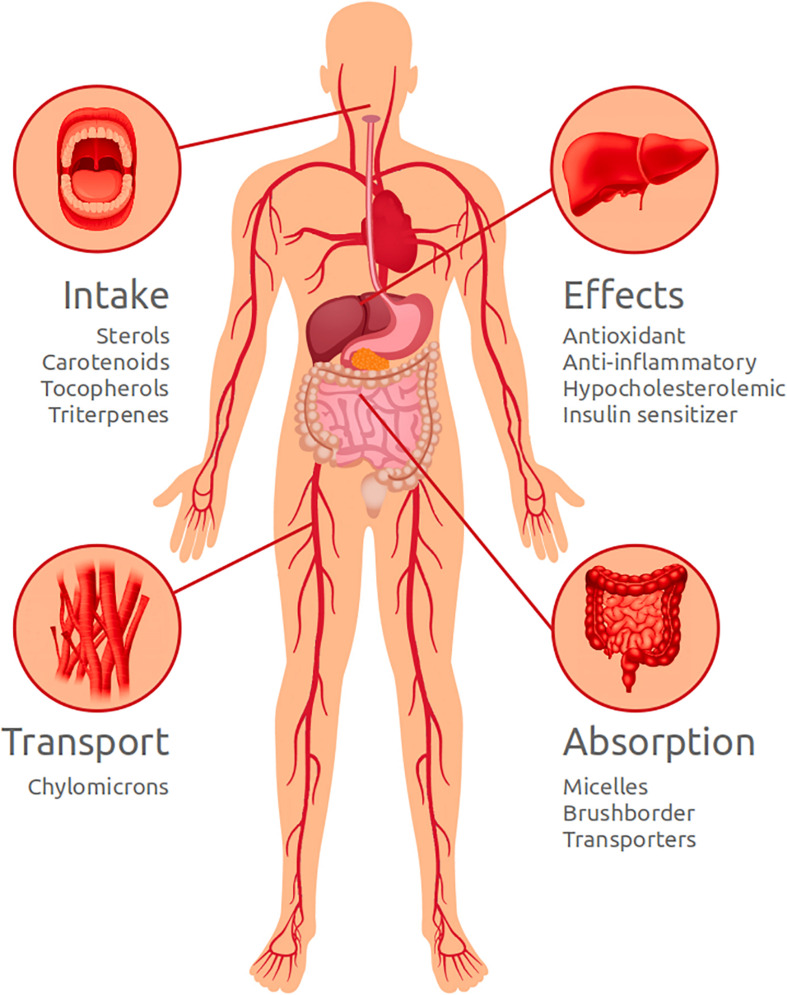
Schematic drawing illustrating the main processes and effects that phytosterol, carotenoids, tocopherols, and triterpenes may have on human body.

Sterols, carotenoids, tocopherols, and pentacyclic triterpenoids are all dietary lipophilic biomolecules with important functional effects for human health. These molecules are solubilized in meal fats and emulsified into mixed micelles in the intestinal lumen, before been taken-up by enterocytes and poured into the bloodstream into chylomicrons. Figure 5 illustrates the main processes and effects that phytosterol, carotenoids, tocopherols, and triterpenes may have on human body.

Although structurally diverse, they share, with different intensity, antioxidant and anti-inflammatory features. By attenuating oxidative stress and inflammation, they improve disorders associated to obesity and dyslipidemia. In animal models and human trials these functional ingredients have demonstrated to decrease plasma levels of TAG, TC, and LDL, whereas arise the leptin, adiponectin and HDL concentrations. They ameliorate hepatic steatosis, protect lipids from oxidation and reduce LDH and MDA productions. These compounds amend BMI, as well as abdominal and subcutaneous obesity.

Furthermore, carotenoids, vitamin E and PT may act as insulin-sensitizers, improving insulin resistance and pathological disorders related to MetSyn. Notoriously, PT also preserve functionality and survival of pancreatic β-cell, increasing the insulin release capability. Vitamin E is a potent lipophilic antioxidant, which scavenge hydroxyl and superoxide radicals and reduce the production of lipid peroxides. PT, by contrast, are modest radical scavengers, but potent enhancers of the adaptive cell response against oxidative and chemotoxic stress. Part of the effects of both tocopherols and PT can be explained by its capability to activate Nrf2 and the expression of phase 2 genes. Through Nrf-2 activation, they upregulate the expression of antioxidant enzymes and lipogenic genes. On the other hand, tocopherols and PT are able to inhibit the transactivation of NFκB, inhibiting the inflammatory response. They repress the production of pro-inflammatory cytokines, the expression of adhesion molecules, and a number of inflammatory pathways, including MAPK, LOX, or COX.

The pharmacological activity of these small lipophilic molecules has been correlated with lower risks to develop T2DM, CVD, and other pathological complications of MetSyn. Unfortunately, accumulated evidence in humans is still limited, and more well-designed RCT should be performed before nutritional recommendations may be directed to general population.

## Author Contributions

All the authors listed have contributed equally, directly and intellectually to the work, and have approved it for publication.

## Conflict of Interest

The authors declare that the research was conducted in the absence of any commercial or financial relationships that could be construed as a potential conflict of interest.
